# Long-term real-life outcomes of the Clareon® hydrophobic intraocular lens: the Clarte study in 191 eyes: 3-years real-life outcomes of the Clareon® intraocular lens

**DOI:** 10.1186/s12886-024-03393-x

**Published:** 2024-03-26

**Authors:** Hugo Bouvarel, Emilie Agard, Jérémy Billant, Antoine Levron, Roman Chudzinski, Hélène Plas, Raphaël Bernier, Lucas Sejournet, Mayeul Chaperon, Corinne Dot

**Affiliations:** 1 Department of Ophthalmology, Hospices Civils de Lyon, E. Herriot University Hospital, Lyon, France; 2 Department of Ophthalmology, Desgenettes Military Hospital, Lyon, France; 3 French Military Medical Academy, Val-de-Grâce, Paris, France

**Keywords:** Cataract surgery, Intraocular lens, Visual acuity, Capsule opacification

## Abstract

**Background:**

To describe and analyze the real-life refractive, functional and safety outcomes of the Clareon® intraocular lens (IOL) after 3 years.

**Methods:**

Data was collected retrospectively for observational purposes between July 2017 and December 2019 in the ophthalmology department of Desgenettes military hospital in Lyon, France. Eyes that underwent cataract surgery with Claeron® implantation were consecutively included. Patients with a systemic or ocular condition that could affect the visual outcome were excluded. Postoperative corrected (CDVA) and uncorrected (UDVA) distance visual acuities as well as capsule and IOL transparency were assessed at 1 month and 3 years.

**Results:**

A total of 326 eyes were analyzed at one month and 191 eyes were reassessed at the 3-year follow-up visit. At 3 years, the mean CDVA was 0.003 LogMAR (95% confidence interval [CI]: -0.003 to -0.01) and the mean UDVA was 0.075 (95% CI: 0.054 to 0.095). Three quarters of the patients had an UDVA ≥ 0.097 logMAR (20/25 Snellen equivalent) and 50% had an UDVA ≥ 0 (20/20). The absence of glistening was reported in 95.3% of cases and 4.7% [9] of patients experienced a clinically significant posterior capsular opacification (PCO) for which Nd:YAG treatment was required.

**Conclusions:**

This real-life study reports high-performance and stable long-term refractive outcomes of the Clareon® IOL with good safety in terms of PCO and glistening.

## Key message

**What is known**

The Clareon® is a recent hydrophobic intraocular lens (IOL) that has shown high-performance functional and safety outcomes in published studies after a 1-year follow-up.

**What is new**

These good functional and safety outcomes were maintained over a 3-year follow-up.

A low rate of Nd:YAG treatment was reported (4.7%), and it was needed late during the third year following surgery. This finding is comparable with what has been reported with the AcrySof material.

A low rate of glistening over time was confirmed.

## Introduction

Cataract is still the leading cause of blindness in high-income countries and in Eastern and Central Europe [1], while technical improvements in surgery and equipment have been made. Surgeons must ensure that the postoperative visual acuity (VA) is maintained over time and the choice of the intraocular lens (IOL) is a major challenge of cataract surgery.

The Clareon® IOL is a recent preloaded 1-piece foldable, aspheric, hydrophobic, monofocal IOL designed by Alcon® and is built on the legacy of the AcrySof® IOL, with a similar design. It is composed of a new hydroxyethyl-methacrylate (HEMA) material with an ultraviolet blocker and a blue light chromophore [2]. The AcrySof® SN60WF model is known for its low posterior capsular opacification (PCO) rate of 4.7% at three years [3], so that it may be used as a comparator. Regarding the Clareon material, several studies have assessed the incidence of PCO and Nd:YAG laser rates in the year following surgery [4]^,^ [5], and only one with a three years follow up recently published [6]. A glistening phenomenon defined as the appearance of water-filled nanovacuoles over time has also been largely associated with the use of hydrophobic material [7], although the consequences on the VA are still debated. Long-term real-life data is missing to assess the efficacy and safety of the Clareon® IOL more than two years after surgery.

The aim of this study was to describe the functional outcomes as well as the IOL and capsule ageing at three years in patients implanted with the Clareon® IOL.

## Materials and methods

### Study design and population

An observational, retrospective, monocentric study was conducted in the ophthalmology department of Desgenettes military hosmital in Lyon (France). Patients who underwent functional cataract surgery with Clareon® CNA0T0 (Alcon Laboratories Inc., Fort Worth, TX, USA) implantation between July 2017 and December 2019 were consecutively included. Some patients attended a 3-year follow-up visit as suggested during the mandatory 1-month postoperative consultation or consulted more often if needed. Patients with a systemic or ocular condition that could affect the visual outcome were excluded, while benign ophthalmological conditions such as mild epiretinal membrane without retraction on en face OCT or early glaucoma were included. Patients with corneal astigmatism greater than 0.75 diopter for whom a toric IOL was needed, pseudo-exfoliation syndrome, an active inflammatory ocular disorder, an active macular disease, and patients experiencing any surgical complications were also excluded. The study adhered to the tenets of the Declaration of Helsinki. Each patient was free to refuse to participate and to oppose to medical data collection according to a specific consent form. This study was approved by the Ethics Committee of the French Society of Ophthalmology (IRB 00008855).

### Surgical procedure

Preoperative biometry using the ZEISS IOLMaster® 700 allowed choosing the Clareon® IOL power using the SRKT formula (A constant of 119.3). The HofferQ and Holladay formulas were also used if the axial length (AL) was less than 22 mm. Phacoemulsification was performed by three senior surgeons using the same surgical protocol through a 2.2-mm main incision using the Alcon Constellation® or Centurion® systems.

### Efficacy outcomes

The primary endpoint was the description of the refractive outcomes at three years. The VA was assessed by senior orthoptists at a distance of 5 m using a decimal scale without any correction (uncorrected distance visual acuity, UDVA) and after subjective refraction (corrected distance visual acuity, CDVA). Patients’ medical records were reviewed to record the best-corrected visual acuity and the UDVA at the mandatory 1-month postoperative consultation and at the final 3-year examination, if patients were not lost to follow-up. All VAs were converted into LogMAR before performing the analyzes. The subjective residual spherical equivalent at one month was also recorded. Some of these patients were also assessed one and two years after surgery, allowing measuring the previously mentioned data at these timepoints and secondarily assessing their stability over time.

### Safety assessment

Safety explorations were considered as secondary descriptions. Patients were clinically assessed by slit-lamp examination by 2 senior surgeons with a double-blind evaluation at the 3-year consultation after full dilation. In case of disagreement a third senior MD was involved (the majority was considered). PCO was graded from 0 when absent, 1 when the proliferation was peripheral, 2 when it was close to the center, to 3 when it reached the center. The number of Nd:YAG capsulotomy procedures already performed was collected at the last 3-year visit and at the subsequent visits if indicated. IOL transparency was assessed using the Miyata’s scale for glistening: grade 0 up to 25 nanovacuoles by mm^2^, grade 1 from 26 to 75 nanovacuolees, grade 2 from 76 to 150 and grade 3 more than 150 nanovacuoles by mm^2^ [8]. The presence of IOL surface haze was also investigated. Other adverse events and surgical complications were also recorded.

### Statistical analysis

Quantitative variables are presented as a mean ± standard deviation and categorical variables as a count (proportion). The VA was compared between the different timepoints for the overall cohort and the subgroups using a non-parametric multiple comparison model using the Kruskal-Wallis test. Patients with missing data were excluded from the analyzes, without data imputation. A Mann-Whitney test was used for the subgroup analyzes. A p-value less than 0.05 was considered statistically significant. All analyzes were performed with Prism software 8.3.1 (GraphPad Software, LLC, San Diego, USA).

## Results

### Primary endpoint: visual acuity at 3 years

A total of 326 eyes of 225 patients were initially included and 191 eyes (59%) of 128 patients were enrolled in the 3-year analysis. A part of patients did not perform any visit at 3 years explaining this number of remaining eyes (*n* = 191) finally evaluated. Patients’ characteristics are shown in Table 1. At 3 years, the mean CDVA was 0.003 LogMAR (95% confidence interval [CI]: -0.003 to -0.01) and the mean UDVA was 0.075 (95% CI: 0.054 to 0.095). Three quarters (73%) of the patients had an UDVA ≥ 0.097 logMAR (20/25 Snellen equivalent) and the UDVA was ≥ 0 (20/20) in 50% of patients.

**Table 1:** Patients’ demographics and ocular characteristics

	Baseline	3 years
**Patients**	*n* = 225	128
age at the time of surgery, years (SD)	73 (7.9)	73 (7.9)
gender, female (%)	120 (53)	73 (38)
both eyes included (%)	102 (31)	59 (46)
**Eyes**	*n* = 326	*n* = 191
right side (%)	154 (47.2)	90 (47.1)
mean AL, mm (SD)	23.8 (1.2)	23.9 (1.1)
mean ACD, mm (SD)	3.18 (0.4)	3.20 (0.40)
mean IOL power	20.5 (2.6)	20.3 (2.7)

SD: standard deviation, VA: visual acuity, AL: axial length, ACD: anterior chamber depth

### Secondary endpoints

#### Safety at 3 years

Among the 191 (59%) eyes examined at 3 years, 9 (4.7%) patients experienced a clinically significant PCO for which Nd:YAG treatment was needed or had already been performed. Central and peripheral PCO were found in respectively 19 (9.9%) and 59 (30.9%) patients. Twenty (10.5%) patients had grade 3 PCO. At the last consultation, 4 (2.1%) patients had already received Nd:YAG treatment during the third year in all cases, and Nd:YAG treatment was planned in 5 (2.6%) other patients after the 3-year visit because of a vision loss.

According to the Miyata’s scale, 95.3% of eyes did not experience glistening at 3 years. Glistening was reported in 8 (4.1%) eyes: 6 (3.1%) were assessed as grade 1 and 2 (1.0%) as grade 2.

### Evolution and parameters measured at other timepoints

**Fig. 1:**
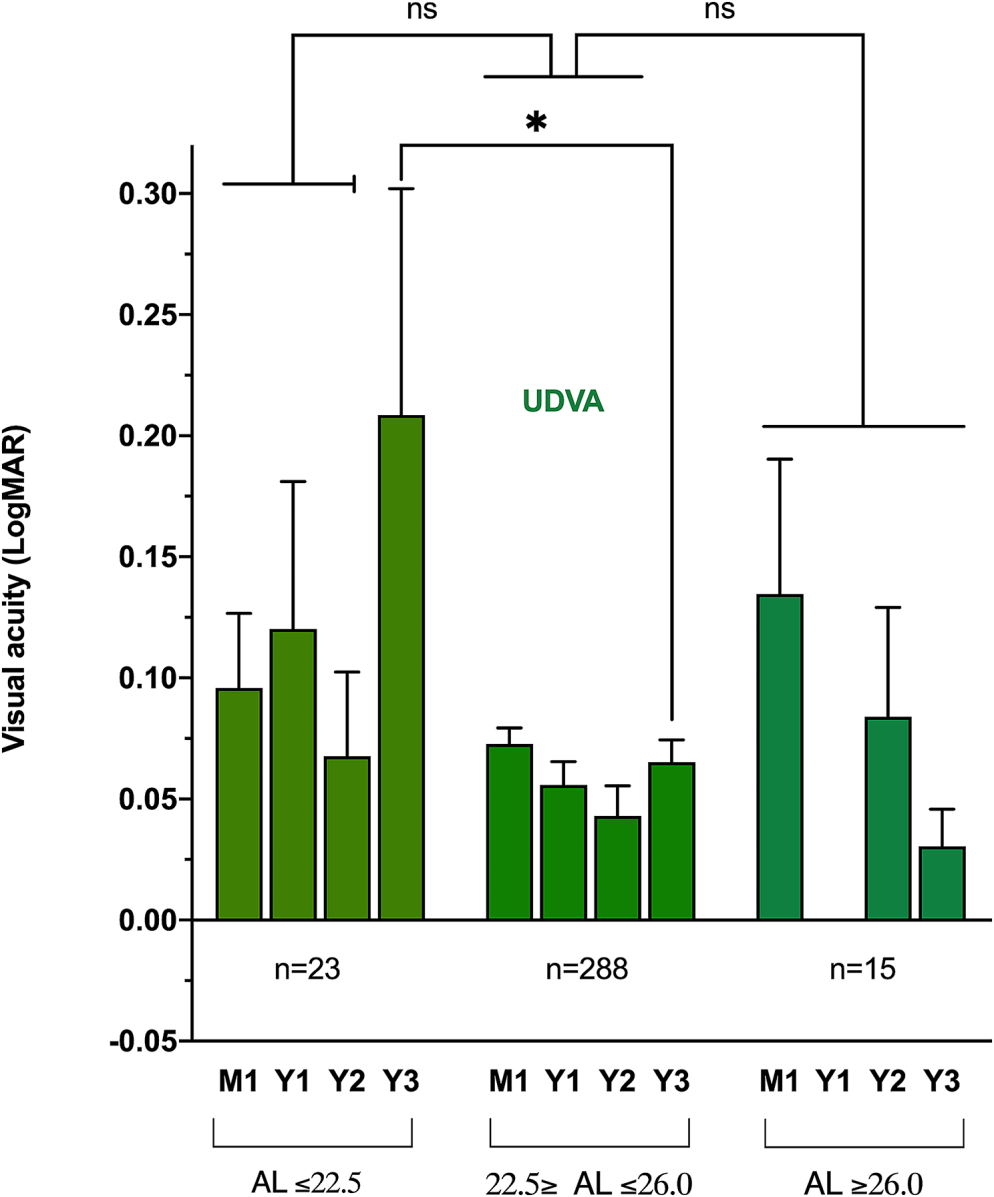
Evolution of the mean corrected and uncorrected distance visual acuities over time in the whole cohort UDVA: uncorrected distance visual acuityCDVA: corrected distance visual acuityns: not significant

The mean CDVA and UDVA did not significantly differ between 1 month and 3 years, with mean differences of respectively − 0.011 (95% CI: -0.021 to 0.0, *p* = 0.16) and 0.0 (95% CI: -0.026 to 0.022, *p* = 0.11). Among the 326 eyes included at baseline, 119 (37%) and 98 (30%) were examined at 1 and 2 years, respectively. Table 2 shows the mean VA and its distribution as well as the rates of PCO and glistening at these timepoints. Figure 1 shows the evolution of the mean VA over time. The comparisons between all the timepoints did not show any significant difference.

**Table 2:** Refractive and safety outcomes over time (n = number of eyes)

	1 month	1 year	2 years	3 years
**Refractive**	*n* = 326	*n* = 119	*n* = 98	*n* = 191
UDVA, LogMAR (SD)	0.077 (0.12))	0.059 (0.097)	0.047 (0.099)	0.075 (0.13)
CDVA, LogMAR (SD)	0.015 (0.057)	0.011 (0.055)	0.01 (0.066)	0.003 (0.045)
**Safety**				
PCO (%)	0 (0)	12 (10)	20 (20)	96 (50)
PCO for which Nd:YAG treatment was needed (%)	0 (0)	0 (0)	0 (0)	9 (4.7)
Glistening (%)	0 (0)	1 (1)	1 (1)	8 (4.1)

UDVA: uncorrected distance visual acuity, CDVA: corrected distance visual acuity, SD: standard deviation, PCO: posterior capsular opacification

### Subgroup analyzes according to the axial length

**Fig. 2:**
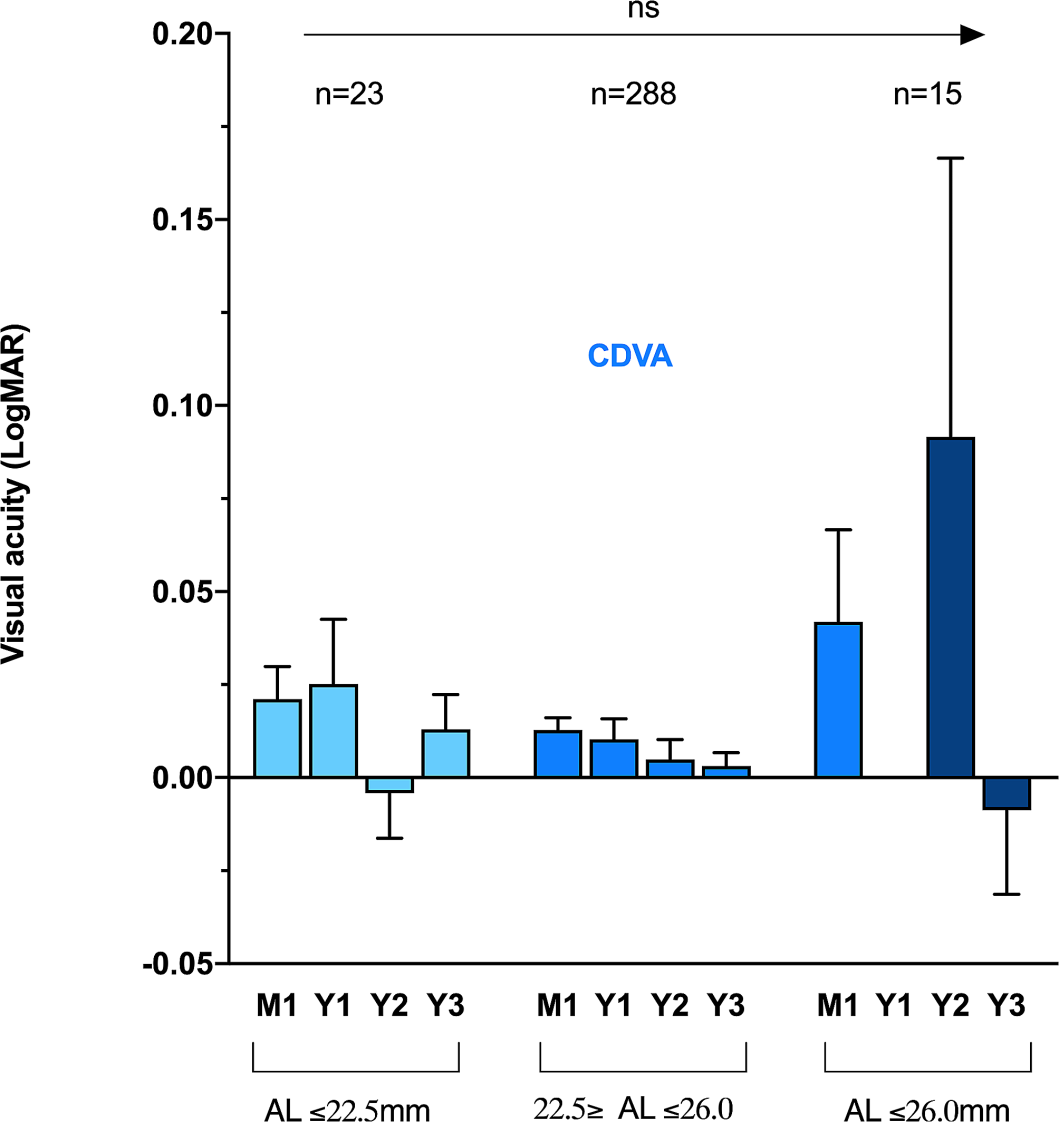
Boxplot showing UDVA distribution and comparison according to the AL subgroups over time UDVA: uncorrected distance visual acuityCDVA: corrected distance visual acuity AL: axial lengthns: not significant n: baseline numbers

**Fig. 3:**
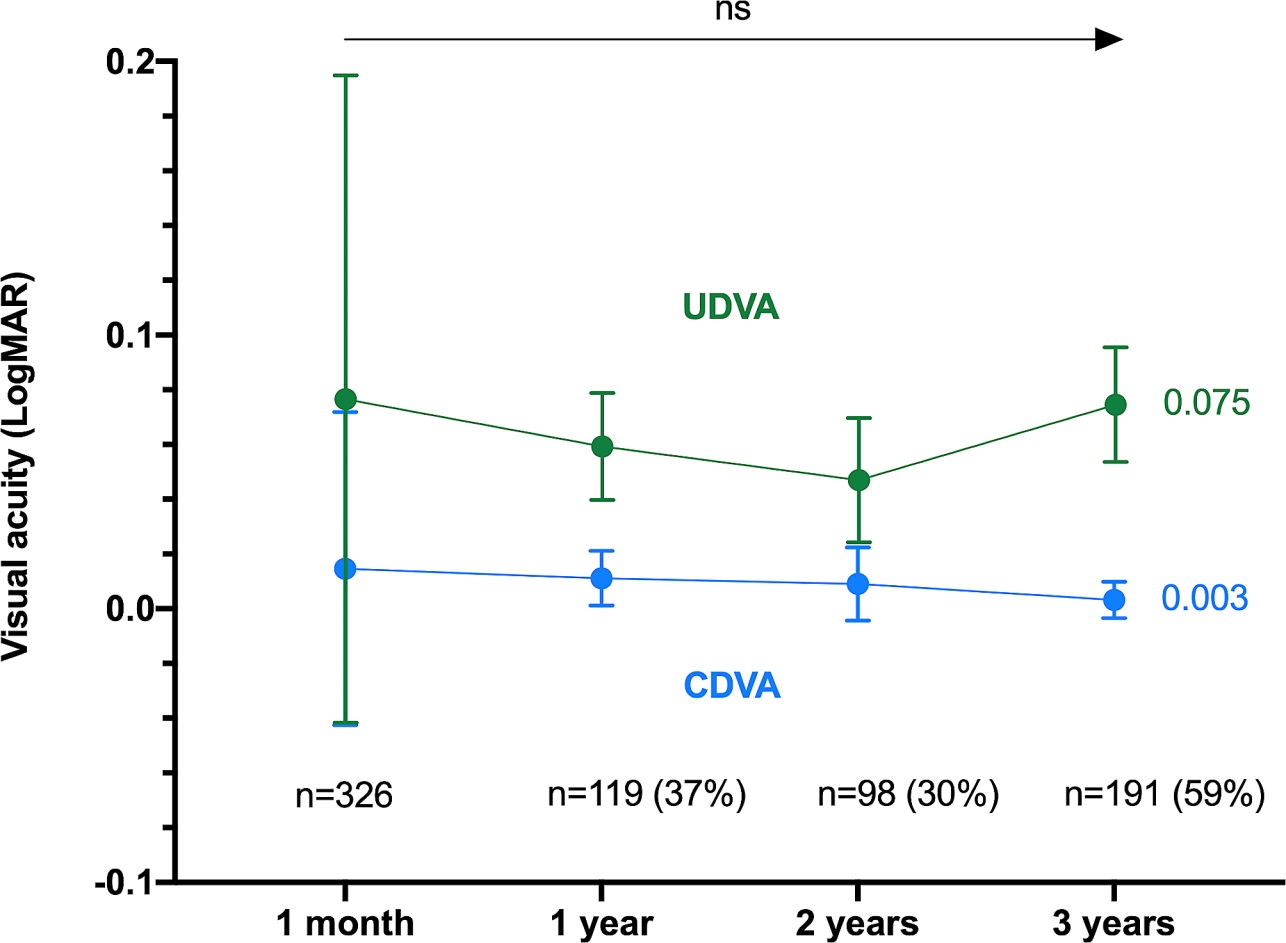
Boxplot showing CDVA distribution and comparison according to the AL subgroups over time UDVA: uncorrected distance visual acuity CDVA: corrected distance visual acuity AL: axial length ns: not significant

Subgroup analyzes were performed according to the AL. Eyes were divided into three groups: AL ≤ 22.5 mm, AL between 22.5 and 26 mm, and AL **≥** 26 mm. No significant difference in CDVA was observed between each group at all the timepoints (Figs. 2, 3).

The UDVA significantly differed between eyes with short and normal AL at 3 years, with respectively 0.21 and 0.065 logMAR (mean difference: -0.14, 95% CI: -0.23 to -0.06, *p* = 0.03) but no significant difference was found between the three subgroups at 1 month, 1 and 2 years. Figures 2 and 3 show the distribution of the VA over time according to the subgroup and their comparison. Within each AL subgroups, the UDVA did not significantly differ over time (respectively *p* = 0.49, *p* = 0.09 and *p* = 0.61). The same stability over time was also found for the CDVA in the three groups (*p* = 0.58, *p* = 0.20 and *p* = 0.57).

### Subgroup analysis of patients treated with Nd:YAG capsulotomy (*n* = 9)

The analysis of the profile of the 9 patients treated with Nd:YAG capsulotomy showed either a young age, an intraocular disorder not classified as an exclusion criterion at baseline (non-contractile epiretinal membrane, pachyvessels), or an intraocular disorder that occurred after surgery (branch retinal vein occlusion, postoperative macular edema).

The mean AL was 25.1 mm in the Nd:YAG group *versus* 23.77 mm in the non-Nd:YAG group. Due to the small number of patients who underwent capsulotomy, no statistical analysis was performed, and the analysis was only descriptive. At 1 month, the mean UCVA and CDVA were comparable to those found in patients who were not treated with Nd:YAG laser.

### Adverse events

We recorded all adverse events, whether they were related to the surgical procedure or not.

For short term at 1 month, one posterior capsular rupture (compatible with an intrasaccular implantation) (1/326) and one post-operative macular edema were recorded (1/326). No endophthalmitis was observed (0/326). Another post-operative macular edema returned to the service for decreased visual acuity 3 months after the surgery. This patient did not come back at 3 years but he consulted at 2 years and this episode did not impact the functional result.

At 3 years, 1/191 eyes had experienced post-operative macular edema (observed at 1 month). This eye required a Nd:YAG capsulotomy after evaluation at 3 years. No retinal detachment was recorded (0/191). Retinal vein occlusions were reported in 4 eyes (4/191).

## Discussion

In this real-life study, we reported an original 3-year overview of the refractive stability and safety of the Clareon® IOL. In the immediate postoperative time, the mean UDVA (≥ 0.097 logMAR or 20/25 Snellen equivalent) allowed spectacle independence in at least three quarters of the patients for activities at far distances such as driving. This result remained stable over time. The UDVA is poorly described in the literature unlike the CDVA. Nevertheless, this result should be considered when planning refractive cataract surgery, especially in elderly patients who may lose their spectacles and thus experience difficulties. Indeed, Knudtson et al. have reported an association between poorer VAs and an increased risk of falls [9]. For example, some elderly patients may wake up and get up during the night with no need to immediately find their spectacles.

Moreover, at the time of refractive cataract surgery, the UDVA is one of the best biomarkers for efficacy and well-being. Our results are consistent with those of Lehman et al. [10], Stanojcic et al. [11], and Titiyal et al. [12] who have assessed the Clareon® IOL but their follow-up was reduced to 1 year. Regarding the CDVA, a high VA (mean: 0.003 LogMAR) was also maintained at 3 years. Other teams have published similar results with a follow-up ≤ 1 year [13]^,^ [14]^,^ [5]^,^ [10]. Very recently, Nuijts et al. reported similar functional results in a prospective study with a three years follow up [6].

This result highlighted the good tolerance of the Clareon® IOL since it is well known that a PCO can impair the vision in the years following cataract surgery. This point was discussed below.

The subgroup analyzes performed according to the AL were reassuring. No significant differences were found between the short, normal and long ALs, except for the UDVA at 3 years between the short and normal ALs. This difference was not observed for the CDVA. This finding could indicate that short eyes could be more prone to refractive changes at 3 years. Nevertheless, the size of the subgroup was limited, and this specific isolated result needs to be confirmed in a larger sample. The absence of difference in UDVA regardless of the subgroup suggested that, even in small capsular bags (i.e., in case of short AL), the Clareon® IOL was stable and no axial displacement led to refractive errors.

In the three subgroups, the VA was also remarkably stable during the follow-up.

Regarding capsular transparency, no clinically significant PCO was observed in 95.3% of patients at 3 years. In other words, Nd:YAG capsulotomy was only required in 4.7% of patients, in line with the Nd:YAG rate of 4.7% reported by Ursell et al. with the AcrySof IOL in their British large data study [3] and by Nuijts et al. in their international and prospective study assessing the Clareon® material [6]. Maxwell et al. have described a lower capsulotomy rate of 1.4% at 3 years in 138 patients but the design of their study was different, and they have used a three-piece IOL composed of the same Clareon material [15]. These results in terms of transparency are encouraging knowing that a recent register study that has assessed over 6,000 thousand surgical procedures has identified that 4.2% of PCO required Nd:YAG treatment at 3 years for hydrophobis IOLs [16]. This is in contrast with a recent national study based on the claims of 6,210 patients which has recently reported that a third of the patients required Nd:YAG capsulotomy within the 2 years following surgery for all IOLs grouped [17]. The impact of Nd:YAG capsulotomy has been highlighted in this study, reporting that early post-cataract capsulotomy was a main driver for adverse event occurrence, especially for ocular hypertension and macula edema. Thus, the use of Nd:YAG capsulotomy should be reduced and delayed as much as possible in our daily practice. PCO occurrence is multifactorial and the IOL material is known as one of the key triggering factors. Indeed, Ursell et al. have reported that the rate of capsulotomy was 2–3 times higher when hydrophilic materials were used [3]. The good safety of the recent Clareon hydrophobic IOL was confirmed in our study with a low rate of capsulotomy; in addition, no capsulotomy was performed before the third year.

Among the 9 patients who needed Nd:YAG treatment, two were under the age of 60 and five presented with systemic or ophthalmological conditions for which local or general medication was needed such as open-angle glaucoma, epiretinal membrane, branch retinal vein occlusion. A young age and a history of vitrectomy are known risk factors for PCO [18]. Glaucoma has also been recently described as a factor contributing to fibrosis development by Dot et al [17]. Proinflammatory profiles have also been described as a risk factor for PCO [19].

Regarding Clareon lens ageing, no surface haze was noted during the follow-up as recently reported by Titiyal et al [12]. Very few and asymptomatic patients presented with low-grade glistening at 3 years, supporting a safe evolution of the yellow Clareon material. This result is in line with the findings of experimental studies that have shown the superiority of the Clareon IOL over hydrophobic IOLs in terms of glistening rate [2]^,^ [20]. This is also in line with the results of Stanojcic et al. who have described only 4 out of 68 eyes implanted with Clareon IOLs experiencing glistening < 10 nanovacuoles per mm^2^ at 1 year [11]. Nuijts et al. have recently reported a grade 0 glistenings in 100% of the cases at three years [6]. We have also used the Miyata classification in which Grade 0 corresponds to very few microvacuoles (0–25/mm2), a data that has been interpreted as glistening free in the recent papers including ours [6].

The resistance of the Clareon IOL to PCO and glistening could be explained by the modified HEMA material. This material binds less to the proteins of the extracellular matrix. The edge design is quite similar to that of the AcrySof IOL in order to block proliferation [21].

There are other complications that a surgeon must face in the long term (related or not to the IOL), mainly retinal detachment [17], which was not described in our cohort on the 191 eyes analyzed at 3 years (including the patient who experienced the capsular rupture). Post-operative macular edema should also be screened, as its early resolution generally does not impair visual acuity, as in the 2 patients in our study. We cannot rule out that postoperative macular oedema may have been overlooked due to the decrease in the cohort over time.

Our study has some limitations, including its retrospective design. Analyses at 3 years were performed on 59% of operated patients. The design did not allow achieving a stable efficacy over time for all the patients and the samples could not be paired, nevertheless this was not the primary endpoint. Also, the monocular VA was only assessed as usually performed in our daily practice. Binocular results would be interesting and could increase the rate of spectacle independence, which is already high. The 3-year visit was performed at the patients’ discretion as suggested immediately after surgery, and this could have induced a bias that increased the rate of Nd:YAG capsulotomy.

The strengths of this study were its long follow-up and the 3-year collection of data on the Clareon® IOL in a real-life setting with less stringent patient selection and more representative of daily practice compared to prospective and randomized studies. The UDVA was systematically assessed and showed the good refractive outcome of this IOL at the time of refractive cataract surgery, and it confirmed the accuracy of the chosen A constant. Only two senior MD performed the postoperative visits to grade PCO and glistening the same day with a double-blind lecture, according to the Miyata’s classification.

This study reports high-performance long-term refractive and functional outcomes of the Clareon® IOL with good safety in terms of PCO and glistening. These findings suggest that the Clareon® IOL and its material could be an interesting and performing choice for upcoming premium IOLs to be implanted in cataract surgery.

## Data Availability

The dataset generated and analysed during the current study are available from the corresponding author on reasonable request.

## Abbreviations

IOL: intraocular lens
CDVA: corrected distance visual acuity
UDVA: uncorrected distance visual acuity
PCO: posterior capsule opacification
HEMA: hydroxyethyl-methacrylate
AL: axial length
Nd YAG: neodymium-doped yttrium aluminium garnet
VA: visual acuity
