# Correction: Long-term real-life outcomes of the Clareon® hydrophobic intraocular lens: the Clarte study in 191 eyes: 3-years real-life outcomes of the Clareon® intraocular lens

**DOI:** 10.1186/s12886-024-03451-4

**Published:** 2024-04-19

**Authors:** Hugo Bouvarel, Emilie Agard, Jérémy Billant, Antoine Levron, Roman Chudzinski, Hélène Plas, Raphaël Bernier, Lucas Sejournet, Mayeul Chaperon, Corinne Dot

**Affiliations:** 1 Department of Ophthalmology, Hospices Civils de Lyon, E. Herriot University Hospital, Lyon, France; 2 Department of Ophthalmology, Desgenettes Military Hospital, Lyon, France; 3 French Military Medical Academy, Val-de-Grâce, Paris, France

**Correction to: J Occup Med Toxicol 15, 36 (2020)**

10.1186/s12886-024-03393-x

In this article, the given and family names of the authors were transposed as Bouvarel Hugo, Agard Emilie, Billant Jérémy, Levron Antoine, Chudzinski Roman, Plas Hélène, Bernier Raphaël, Sejournet Lucas, Chaperon Mayeul & Dot Corinne.

The author group has been updated above and the original article has been corrected.

